# Prevalence of Crimean-Congo Hemorrhagic Fever Virus in Healthy Population, Livestock and Ticks in Kosovo

**DOI:** 10.1371/journal.pone.0110982

**Published:** 2014-11-13

**Authors:** Luka Fajs, Isme Humolli, Ana Saksida, Nataša Knap, Mateja Jelovšek, Miša Korva, Isuf Dedushaj, Tatjana Avšič-Županc

**Affiliations:** 1 Institute of Microbiology and Immunology, Faculty of Medicine, University of Ljubljana, Ljubljana, Slovenia; 2 National Institute of Public Health in Kosovo, Pristina, Kosovo; Division of Clinical Research, United States of America

## Abstract

Crimean-Congo hemorrhagic fever (CCHF) is an acute, tick borne disease often associated with hemorrhagic presentations and high case fatality rate. Kosovo is a highly endemic area for CCHF, with a significant case fatality rate. The aim of our study was to determine the prevalence of CCHF in Kosovo. We tested 1105 serum samples from healthy population in both endemic and non-endemic areas in the country. Our results revealed a seroprevalence of 4.0% (range 0–9.3%) which is comparable to the seroprevalence in other countries. We show that seroprevalence is correlated to the disease incidence in each studied municipality. We also tested 401 animal sera (353 cow, 30 sheep, 10 goat and 8 chicken) in four endemic municipalities in Kosovo. We detected specific antibodies in all animals except in chicken. Seroprevalence in cows is comparable to other endemic areas and correlates to the seroprevalence in humans. No CCHF RNA could be detected in 105 tick samples obtained in 2012 and 2013. Sequencing of CCHFV positive ticks from 2001 revealed that the virus is most closely related to viral strains that were detected in CCHF patients from Kosovo. Results suggest that mild CCHF cases are most probably underdiagnosed and consequently that the burden of disease is higher than reported. Our study provides key information for CCHF surveillance and raises awareness for possible imported cases in CCHF non-endemic countries.

## Introduction

Crimean-Congo hemorrhagic fever is an emerging tick-borne zoonotic disease often associated with hemorrhagic presentations and a case fatality rate of 10–50%. Causative agent is the Crimean-Congo hemorrhagic fever virus (CCHFV) (genus *Nairovirus*, family *Bunyaviridae*), the most widespread tick-borne virus in the world. CCHF cases have been reported in Africa, south-eastern Europe, Middle East, and Asia, with significant increase in disease incidence in the last decade, especially in the south-eastern Europe [1].

Kosovo, a south-eastern European country, is an endemic area for CCHF. It covers an area of approximately 11,000 km^2^ with a population of 1.9 million. The endemic zone, which represents half of the Kosovo territory, is characterized by a hot and dry climate, low bushes and high density of agriculture and farming. Climate is influenced by continental air masses resulting in relatively cold winters and hot and dry summer and autumn. These conditions provide an ideal ecosystem for *Hyalomma* ticks which are the main vector of CCHFV [2], [3].

CCHF has been present in Kosovo for at least a half of century. The first documented cases date back to 1957, when seven fatal cases were reported. Since then, CCHF cases emerged sporadically until 1995 when 46 confirmed cases were reported, with 7 fatal cases [4]. Based on the records of the Institute of Public Health of Kosovo, from 1995 to August 2013, the number of confirmed cases rose to 228, with 59 fatalities (Table 1). The causative strain of CCHF in Kosovo is CCHFV Hoti, which was isolated during the 2001 epidemic [5]. Case fatality rate of CCHF infections in Kosovo is on average 25.5% [4]. This case fatality rate is much higher than the reported case fatality rate in Turkey (5–10%), another highly endemic country in vicinity [6]. The reasons for the observed difference in the case fatality rates has never been determined or studied. Possible explanation for this could be a lower rate of diagnosis of mild and asymptomatic cases in Kosovo, difference in virulence of the causative virus strains or host immune genetic differences in the populations. Because of the yet unexplained high case fatality rate and the increasing incidence of CCHF, Kosovo represents a hot spot for deadly CCHF infections and can serve as a model region for studies of disease prevalence and spread. This importance is heightened because of its vicinity to other European countries where no cases have yet been reported but do have competent tick vectors [7].

**Table 1:** CCHF case statistics from 1995 to August 2013.

Year	Cases	Deaths	Incidence (per 100,000)	CFR† [%]
1995	46	7	2.29	15.21
1996	9	5	1.04	21.70
1997	0	0	0	0.00
1998	1	0	0.04	0.00
1999	3	2	0.3	28.60
2000	1	0	0.09	0.00
2001	31	7	1.3	22.60
2002	14	3	0.57	21.40
2003	6	3	0.4	50.00
2004	16	2	1.04	12.50
2005	11	5	0.68	45.40
2006	5	2	0.3	40.00
2007	2	2	0.12	100.00
2008	5	1	0.41	14.30
2009	10	3	0.81	21.40
2010	29	7	1.2	24.10
2011	5	0	0.3	0.00
2012	12	2	0.63	16.70
2013	22	8	1.57	36.40
Total	228	59	0.69	25.50

† CFR =  case fatality rate.

Whereas clinical cases have been extensively studied in previous studies, there is limited knowledge about the overall seroprevalence of the disease in Kosovo [4], [8]–[10]. For this reason, the aim of our study was to determine the (sero)-prevalence of CCHFV in general population and animals in Kosovo.

## Methods

### CCHF seroprevalence in general population of Kosovo

We included 1105 serum samples from residents (46% male) of 15 Kosovo municipalities (both endemic and non-endemic). Samples were obtained from May to December 2012 from residents administered to primary health-care centers for blood-testing with no signs of infectious disease illness. Sampling was therefore not targeted to individuals with a higher risk of infection (eg. individuals reporting a tick-bite) but to a general population. Median age of participants was 45 years (range 0–85 years). Distribution of samples in different age groups was comparable: 126 (<20 y/o.), 161 (20–30 y/o), 163 (30–40 y/o), 208 (40–50 y/o), 182 (50–60 y/o), 142 (60–70 y/o) and 113 (>70 y/o).

Samples were tested for the presence of anti- CCHFV IgG antibodies with VectoCrimea - CHF - IgG ELISA (Vector Best, Novosibirsk, Russia). We chose VectoCrimea - CHF - IgG ELISA because of its high sensitivity, specificity and the overall high score as evaluated in a multicenter study of CCHF diagnostic tests, by a working group of experts from reference laboratories under the initiative of the European Network for Diagnostics of Imported Viral Diseases [11] and to allow better comparison to other similar studies [16]–[17].

Research was approved by the National Medical Ethics Committee of the Republic of Kosovo, protocol number 05-3193/2. National Medical Ethics Committee of the Republic of Kosovo waived the need of a written informed consent, but oral informed consent was obtained from all donors or the next of kin. We followed the principles of the Helsinki Declaration, the Oviedo Convention on Human Rights and Biomedicine, and the Slovene Code of Medical Deontology. All human samples were anonymized and no additional sample was taken for the purpose of the study.

### CCHF seroprevalence in livestock

We investigated 401 animal sera (353 cow, 30 sheep, 10 goat and 8 chicken) that were collected from June to November 2012 in four endemic municipalities in Kosovo: Malishevë, Rahovec, Klinë and Suharekë. Samples were taken from animals in randomly selected villages, by local veterinary specialists. National Medical Ethics Committee of the Republic of Kosovo waived the need of a written informed consent or specific ethics approval because the samples were primarily taken for routine diagnostic surveillance by the local veterinary specialists. Animal owners provided oral informed consent and gave their permission for the collection of animal sera and their use in the study. All samples and personal data were anonymized and no additional samples were taken for the purpose of the study. Animal sera were tested with an in-house immunofluorescence assay (IFA) using CCHFV strain Hoti-infected Vero E6 cells, fixed on microscope slides. Bovine antibodies were detected with Anti-Bovine IgG (whole molecule)–FITC antibody (Sigma), sheep antibodies were detected with rabbit Anti-Sheep IgG (H+L)–FITC antibody (KPL), goat antibodies were detected with Anti-Goat IgG (whole molecule)–FITC antibody (Sigma), chicken antibodies were detected with Anti-Chicken IgG (whole molecule)–FITC antibody (Sigma). Samples were initially diluted 1∶10. Positive samples were further diluted to 1∶320.

### CCHF prevalence in ticks

We collected 105 adult ticks from infested livestock in three hyper-endemic municipalities (Malishevë, Rahovec and Klinë) between 2012 and 2013. Ticks were processed as described by Durmisi et al. [12]. Total RNA from tick homogenates was extracted using Trizol Reagent (Invitrogen Life Technologies) according to the manufacturer's instructions. Presence of CCHFV RNA was determined and quantified by a quantitative Real-Time RT-PCR protocol described by Duh et al. [13]. Real-Time RT-PCR positive samples were further processed with a nested RT-PCR protocol described by Rodriguez et al. [14] which produces a 260 bp fragment of the CCHFV S segment. RT-PCR was performed using the SuperScript III One-Step RT-PCR System with Platinum Taq (Invitrogen Life Technologies) according to the manufacturer's instructions.

Additionally, we included 5 CCHFV RNA positive tick-pool samples reported by Duh et al [13], which were processed as described above, for sequencing and phylogenetic analyses.

### Sequencing and nucleotide sequence analysis

PCR products were purified with the Wizard SV Gel and PCR Clean-Up System (Promega), sequenced using the BigDye Terminator 3.1 Cycle sequencing kit (Applied Biosystems) and analyzed with the 3500 Genetic Analyzer (Applied Biosystems).

Nucleotide sequences were assembled and edited using CLC Main Workbench software (CLC bio, Denmark). At least two-fold read coverage was obtained for all sequences. Sequences were aligned in MEGA version 6 using Muscle algorithm [15]. Nucleotide sequences were deposited to the GenBank database. Nucleotide substitution model (GTR+G+I) was selected based on Akaike's information criterion (AIC) in jModelTest, version 2.1.4 [16]. Maximum likelihood phylogenetic analysis (with 500 bootstrap replicates) and depiction of phylogenetic trees was performed in MEGA version 6 [15].

## Results and Discussion

**Figure 1:**
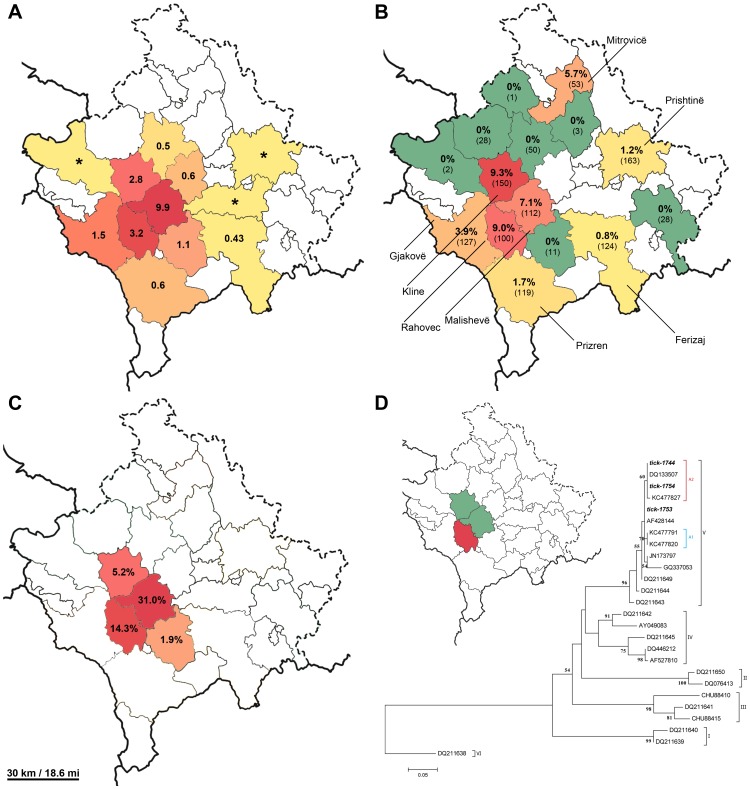
Prevalence of CCHF in Kosovo A. Cumulative incidence (per 100,000) of CCHF (from 1995 to 2013) in each municipality of Kosovo. Asterisk (*) represents a single reported CCHF case. **B.** Seroprevalence of CCHF in healthy human population in the studied municipalities of Kosovo and the number of tested sera in each municipality (in brackets), **C.** CCHF seroprevalence in cows in the studied municipalities of Kosovo, **D.** maximum likelihood phylogenetic analysis (with 1000 bootstrap) of partial (260 bp) CCHFV S segment nucleotide sequences. The map shows the municipalities where ticks were collected (green and red) and the municipality (red) of the positive ticks that were included in the study for CCHFV sequencing. Designations A1 and A3 represent Kosovo CCHFV sub-lineages described by Fajs et al. [27].

Forty-four participants (4.0%) had detectable IgG antibodies to CCHFV. The highest seroprevalence was observed in the hyper-endemic regions of Klinë (9.3%), Rahovec (9.0%) and Malishevë (7.1%) (Figure 1A). Overall seroprevalence is comparable to other endemic countries in the Balkan region, Bulgaria (2.8%) [16], Greece (4.2%) [18] and Turkey (2.3%) [19] and notably lower than in earlier reports from Turkey (10–19.6%) [20]–[22]. Rather higher seroprevalence in these reports reflects the extreme rise in CCHF incidence in Turkey in the last 10 years [6] and probably also the differences in sampling. Namely, only highly CCHF endemic areas were included in these studies in Turkey. Thereby, seroprevalence in the hyper-endemic areas in Kosovo is comparable to the reports from Turkey. Since the overall seroprevalence rates in both countries are comparable, our results suggest that there is a high rate of unapparent infections in Kosovo. In this view the burden of CCHF is most probably even higher than reported.

Seroprevalence in Kosovo is generally consistent with cumulative incidence of CCHF (from year 1999 to 2013) in different municipalities (Figure 1B), with highest seroprevalence determined in areas with highest incidence of the disease. However, there is one exception of note; that is the seroprevalence in Mitrovicë municipality, where no cases were reported to date. In order to explain the situation we interviewed all serologically positive participants and none reported a tick bite in the recent past or travel to endemic zones. These results suggest that CCHFV could have been present in this region in the past and has not been recognized. Of note are also municipalities neighboring the hyper-endemic regions (Klinë, Rahovec and Malishevë) with no CCHF seroprevalence. The absence of IgG positives is most likely due to the low sampling numbers and the low seroprevalence of CCHF in these areas.

Demographically, the majority of serologically positive participant were male (69.7%), 60–70 years old (median 62 years). Mean age of seropositive participants is slightly higher than in previous report and reports from other countries in the region [2, 4, 9_ENREF_8, 16_ENREF_10, 20]. These results indicate that CCHF has been present in Kosovo for a long time. This could also be attributable to the fact that this population was mostly involved with agriculture and therefore at higher risk of coming into contact with infected ticks.

CCHFV specific IgG antibodies were detected in 65 (18.4%) cows, 2 (20%) goats, 3(10%) sheep (Table 2). Seroprevalence in cows was highest in Malishevë and Rahovec municipalities (31.0% and 14.3% respectively). This is consistent with the highest observed seroprevalence in healthy human population as well as with the incidence rates of CCHF in Kosovo. In general, the seroprevalence in animals is comparable to the observed seroprevalence in livestock in other endemic countries [23]–[26].

**Table 2:** Seroprevalence in livestock from Kosovo.

		Municipality					
animal	IgG	Rahovec	Malishevë	Prizren	Klinë	Suharekë	Total
cow	pos	12 (14.3%)	49 (31.0%)	/	3 (5.2%)	1 (1.9%)	65 (18.4%)
	+/−	3 (3.6%)	4 (2.5%)	/	1 (1.7%)	/	8 (2.3%)
	neg	69 (82.1%)	105 (66.5%)	/	54 (93.1%)	52 (98.1%)	280 (79.3%)
	total	84	158	/	58	53	353
goat	pos	/	2 (20%)	/	/	/	2 (20%)
	+/−	/	1 (10%)	/	/	/	1 (10%)
	neg	/	7 (70%)	/	/	/	7 (70%)
	total	/	10	/	/	/	10
sheep	pos	/	3 (10.3%)	/	/	/	3 (10%)
	neg	/	26 (89.7%)	/	1 (100%)	/	27 (90%)
	total	/	29	/	1	/	30
chicken	pos	/	/	/	/	/	/
	neg	2 (100%)	4 (100%)	2 (100%)	/	/	8 (100%)
	total	2	4	2	/	/	8
	Total	86	201	2	59	53	401

Chicken were included in the study because while performing collection of ticks in endemic areas, we observed that farms that harbor chicken had no or only mild tick infestation compared to neighboring farms with high tick infestation and no poultry. We assume that the reason for this observation is the ingestion of the ticks by chicken. No anti-CCHFV antibodies could be detected in chicken, however the sample size was very small. Therefore further efforts should be put out to determine the role of chicken in containment of tick populations in endemic areas and their serological status regarding CCHFV.

Furthermore, we analyzed 105 tick samples for the presence of CCHFV RNA that were collected from livestock in the hyper-endemic areas of Malishevë, Rahovec and Klinë. We could not detect viral RNA in any sample. The absence of detectable CCHFV RNA in ticks is somewhat surprising since a previous report showed a 15% prevalence of CCHFV RNA in ticks in Kosovo, in the same region [13]. This absence could be explained by the fact that the sampling was carried out soon after the stables were treated with acaricides. Also, a larger sample size should be included in future studies. In order to explore genetic variability of CCHFV in ticks in Kosovo, we then analyzed the CCHFV RNA-positive ticks reported by Duh et al. [13]. We obtained partial S segment sequences from three tick samples. Phylogenetic analysis revealed, that the tick-derived CCHFV sequences clustered in the European clade I, together with other sequences from Kosovo (Figure 1D). Furthermore, the analysis revealed that two sequences clustered into a previously described Kosovo sub-lineage A1, which includes the prototype Kosovo CCHFV strain Hoti and its variants that were detected in CCHF patients [27]. The other sequence was closely related to the A1 sub-lineage. These results show, that tick-derived sequences from the endemic area are closely related to the patient-derived sequences in Kosovo. However, further studies are necessary in order to determine a broader spectrum of genetic diversity of CCHFV in ticks in Kosovo.

The results of our study clearly show an overlap between the incidence rates of CCHF in Kosovo, seroprevalence in healthy human population and the seroprevalence in animals. From an ecological point of view, the results are consistent with the ecological niches present in Kosovo. Seroprevalence is the highest in regions with low vegetation and high density of farming. In these areas the main occupation is farming and animal breeding. Agriculture in these areas is limited to individual small farms and people commonly get infected by a tick bite while in the pasture. On the other hand, areas of low seroprevalence are mostly wooded areas or areas of high altitude.

Our study provides a comprehensive view of CCHFV (sero)-prevalence in Kosovo, an important endemic area for CCHF. High density of farming and agriculture in combination with favorable climate conditions make it an ideal ecosystem for CCHFV. Seroprevalence results revealed CCHF outbreak hot spots and will enable a more focused preventive approach towards limiting the spread of this emerging disease in Kosovo. Our study shows that Kosovo is an important region for CCHF and raises awareness for diagnostics of imported cases in countries where CCHF is not endemic. In our view, further surveillance of disease prevalence and spread into new territories should be carefully monitored in the future.

## References

[1] Bente DA, Forester NL, Watts DM, McAuley AJ, Whitehouse CA, et al (2013) Crimean-Congo hemorrhagic fever: History, epidemiology, pathogenesis, clinical syndrome and genetic diversity. Antiviral Res 100(1): 159–189. 23906741 10.1016/j.antiviral.2013.07.006

[2] Hoogstraal H (1979) The epidemiology of tick-borne Crimean-Congo hemorrhagic fever in Asia, Europe, and Africa. J Med Entomol 15(4): 307–417. 113533 10.1093/jmedent/15.4.307

[3] Avsic-Zupanc T (2007) Epidemiology of Crimean-Congo hemorrhagic fever in the Balkans. In: Ergonul O, Whitehouse CA, editors. Crimean-Congo Hemorrhagic Fever: A Global Perspective. Dordrecht: Springer. 328.

[4] Humolli I, Dedushaj I, Zupanc TA, Mucaj S (2010) Epidemiological, serological and herd immunity of Crimean-Congo hemorrhagic fever in Kosovo. Med Arh 64(2): 91–93. 20514773

[5] Duh D, Nichol ST, Khristova ML, Saksida A, Hafner-Bratkovic I, et al (2008) The complete genome sequence of a Crimean-Congo hemorrhagic fever virus isolated from an endemic region in Kosovo. Virology J 5: 7. 18197964 10.1186/1743-422X-5-7 PMC2266736

[6] Dokuzoguz B, Celikbas AK, Gök SE, Baykam N, Eroglu MN, et al (2013) Severity scoring index for Crimean-Congo hemorrhagic fever and the impact of ribavirin and corticosteroids on fatality. Clin Infect Dis 57(9): 1270–1274. 23946218 10.1093/cid/cit527

[7] Estrada-Pena A, Jameson L, Medlock J, Vatansever Z, Tishkova F (2012) Unraveling the ecological complexities of tick-associated Crimean-Congo hemorrhagic fever virus transmission: a gap analysis for the western palearctic. Vector Borne Zoonotic Dis 12(9): 743–752. 22448676 10.1089/vbz.2011.0767

[8] Duh D, Saksida A, Petrovec M, Dedushaj I, Avsic-Zupanc T (2006) Novel one-step real-time RT-PCR assay for rapid and specific diagnosis of Crimean-Congo hemorrhagic fever encountered in the Balkans. J Virol Methods 133(2): 175–179. 16343650 10.1016/j.jviromet.2005.11.006

[9] Duh D, Saksida A, Petrovec M, Ahmeti S, Dedushaj I, et al (2007) Viral load as predictor of Crimean-Congo hemorrhagic fever outcome. Emerg Infect Dis 13(11): 1769–1772. 18217568 10.3201/eid1311.070222 PMC3375790

[10] Saksida A, Duh D, Wraber B, Dedushaj I, Ahmeti S, et al (2010) Interacting roles of immune mechanisms and viral load in the pathogenesis of crimean-congo hemorrhagic fever. Clin Vaccine Immunol 7(7): 1086–1093. 10.1128/CVI.00530-09 PMC2897258 20484568

[11] Vanhomwegen J, Alves MJ, Avsic-Zupanc T, Bino S, Chinikar S, et al (2012) Diagnostic assays for Crimean-Congo hemorrhagic fever. Emerg Infect Dis 18(12): 1958–1965. 23171700 10.3201/eid1812.120710 PMC3557897

[12] Durmisi E, Knap N, Saksida A, Trilar T, Duh D, et al (2011) Prevalence and molecular characterization of tick-borne encephalitis virus in Ixodes ricinus ticks collected in Slovenia. Vector borne and zoonotic diseases 11: 659–664. 21028962 10.1089/vbz.2010.0054

[13] Duh D, Saksida A, Petrovec M, Dedushaj I, Avsic-Zupanc T (2006) Novel one-step real-time RT-PCR assay for rapid and specific diagnosis of Crimean-Congo hemorrhagic fever encountered in the Balkans. J Virol Methods 133: 175–179. 16343650 10.1016/j.jviromet.2005.11.006

[14] Rodriguez LL, Maupin GO, Ksiazek TG, Rollin PE, Khan AS, et al (1997) Molecular investigation of a multisource outbreak of Crimean-Congo hemorrhagic fever in the United Arab Emirates. Am J Trop Med Hyg 57: 512–518. 9392588 10.4269/ajtmh.1997.57.512

[15] Tamura K, Stecher G, Peterson D, Filipski A, Kumar S (2013) MEGA6: Molecular Evolutionary Genetics Analysis version 6.0. Molecular Biology and Evolution 30: 2725–2729. 24132122 10.1093/molbev/mst197 PMC3840312

[16] Darriba D, Taboada GL, Doallo R, Posada D (2012) jModelTest 2: more models, new heuristics and parallel computing. Nat Methods 9: 772. 10.1038/nmeth.2109 PMC4594756 22847109

[17] Christova I, Gladnishka T, Taseva E, Kalvatchev N, Tsergouli K, et al (2013) Seroprevalence of Crimean-Congo hemorrhagic fever virus, Bulgaria. Emerg Infect Dis 19(1): 177–179. 23260369 10.3201/eid1901.120299 PMC3557978

[18] Sidira P, Maltezou HC, Haidich AB, Papa A (2012) Seroepidemiological study of Crimean-Congo hemorrhagic fever in Greece, 2009–2010. Clin Microbiol Infect 18(2): E16–E19. 22192082 10.1111/j.1469-0691.2011.03718.x

[19] Yagci-Caglayik D, Korukluoglu G, Uyar Y (2004) Seroprevalence and risk factors of Crimean-Congo hemorrhagic fever in selected seven provinces in Turkey. J Med Virol 86: 306–314. 10.1002/jmv.23699 24037814

[20] Koksal I, Yilmaz G, Aksoy F, Erensoy S, Aydin H (2013) The seroprevalance of Crimean-Congo hemorrhagic fever in people living in the same environment with Crimean-Congo hemorrhagic fever patients in an endemic region in Turkey. Epidemiol Infect 21: 1–7. 10.1017/S0950268813001155 PMC9151165 23688370

[21] Ertugrul B, Kirdar S, Ersoy OS, Ture M, Erol N, et al (2012) The seroprevalence of Crimean-Congo hemorrhagic fever among inhabitants living in the endemic regions of Western Anatolia. Scand J Infect Dis 44(4): 276–281. 22017179 10.3109/00365548.2011.621445

[22] Bodur H, Akinci E, Ascioglu S, Onguru P, Uyar Y, et al (2012) Subclinical infections with Crimean-Congo hemorrhagic fever virus, Turkey. Emerg Infect Dis 18(4): 640–642. 22469474 10.3201/eid1804.111374 PMC3309668

[23] Gergova I, Kamarinchev B (2013) Comparison of the prevalence of Crimean-Congo hemorrhagic fever virus in endemic and non-endemic Bulgarian locations. J Vector Borne Dis 50(4): 265–270. 24499848

[24] Mostafavi E, Haghdoost A, Khakifirouz S, Chinikar S (2013) Spatial analysis of Crimean Congo hemorrhagic fever in Iran. Am J Trop Med Hyg 89(6): 1135–41.. 24166038 10.4269/ajtmh.12-0509 PMC3854891

[25] Adam IA, Mahmoud MA, Aradaib IE (2013) A seroepidemiological survey of Crimean Congo hemorrhagic fever among cattle in North Kordufan State, Sudan. Virol J 10: :178. 10.1186/1743-422X-10-178 PMC3679941 23738961

[26] Mourya DT, Yadav PD, Shete AM, Gurav YK, Raut CG, et al (2012) Detection, isolation and confirmation of Crimean-Congo hemorrhagic fever virus in human, ticks and animals in Ahmadabad, India, 2010–2011. PLoS Negl Trop Dis 6(5): e1653. 22616022 10.1371/journal.pntd.0001653 PMC3352827

[27] Fajs L, Jakupi X, Ahmeti S, Humolli I, Dedushaj I, et al (2014) Molecular epidemiology of Crimean-Congo hemorrhagic fever virus in Kosovo. PLoS Negl Trop Dis 9 8(1): e2647. 10.1371/journal.pntd.0002647 PMC3886908 24416468

